# Early In-Hospital Initiation of Sodium–Glucose Cotransporter-2 Inhibitors After ST-Elevation Myocardial Infarction: A Clinical Review of Hemodynamic and Renal Safety

**DOI:** 10.31083/RCM48560

**Published:** 2026-04-22

**Authors:** Muhammad Farhan, Tirath Patel, Dania Almarouj, Ariana Seyfi, Syed Imran Ali Abdi, Abdulrahman Abufanas, Aya Rimawi, Ibrahim Al-Zuhairi, Ahmed Al-Zuhairi, Abdullah Al Azzawi, Bhumi Daishik Patel, Ayoola Awosika

**Affiliations:** ^1^Internal Medicine, College of Medicine, Ajman University, 6263 Ajman, United Arab Emirates; ^2^Internal Medicine, School of Medicine, Trinity Medical Sciences University, VC 01000 Kingstown, Saint Vincent and the Grenadines; ^3^Internal Medicine, Faculty of Medicine and Healthcare, Al-Farabi Kazakh National University, 050040 Almaty, Kazakhstan; ^4^Internal Medicine, Thumbay University Hospital, 6263 Ajman, United Arab Emirates; ^5^Internal Medicine, College of Medicine, University of Sharjah, 6263 Sharjah, United Arab Emirates; ^6^Internal Medicine, Windsor University School of Medicine, 00265 Basseterre, Saint Kitts and Nevis; ^7^Department of Family and Community Medicine, University of Illinois College of Medicine, Peoria, Bloomington, IL 61701, USA

**Keywords:** ST-elevation myocardial infarction, myocardial infarction, sodium–glucose transporter-2 inhibitors, acute kidney injury, acute coronary syndrome

## Abstract

Survivors of ST-elevation myocardial infarction (STEMI) continue to face appreciable risks of hospitalization for heart failure and acute kidney injury (AKI), even in the era of prompt primary percutaneous coronary intervention (PCI). Sodium–glucose cotransporter-2 inhibitors (SGLT2i) deliver proven cardio–renal benefits in chronic heart failure and chronic kidney disease (CKD); however, the safety profile of these agents when initiating administration during the index STEMI admission remains poorly characterized. This clinical review summarizes contemporary evidence on the hemodynamic and renal safety of initiating SGLT2i therapy 24–72 h after PCI in patients with STEMI and provides a pragmatic, evidence-informed bedside framework, supported by randomized controlled trials (RCTs) and mechanistic and observational data from January 2018 to July 2025. Trial eligibility criteria and safety endpoints were extracted qualitatively; no formal meta-analysis was performed. Among at least 11,221 participants, early SGLT2i initiation was well tolerated: rates of hypotension, volume depletion, AKI, and diabetic ketoacidosis (DKA) were comparable to placebo. The characteristic 3–6 mL/min/1.73 m^2^ decline in estimated glomerular filtration rate (eGFR) represented a reversible tubuloglomerular feedback (TGF) adjustment rather than nephrotoxicity. Mechanistic studies attribute these findings to mild natriuresis without sympathetic activation and to afferent arteriolar vasoconstriction, which lowers intraglomerular pressure. Synthesizing trial exclusion criteria with clinical judgement, we propose the START checklist (stable hemodynamics, tubular reserve, acid–base stability, risk factors, timing (24–72 h)) as provisional guidance to support bedside decision-making while large outcome studies, such as the empagliflozin after acute myocardial infarction (EMPACT-MI) trial and the extended empagliflozin to prevent worsening of left ventricular volumes and systolic function after myocardial infarction (EMPRESS-MI) trial read-outs, are awaited. Current evidence supports the hemodynamic and renal safety of commencing SGLT2i soon after PCI in hemodynamically stable STEMI patients with preserved tubular reserve. In the absence of ongoing trials, cautious adoption guided by the START framework can help clinicians capture potential cardio–renal benefits without compromising acute care.

## 1. Introduction

ST-elevation myocardial infarction (STEMI) survivors face substantial risk of complications even with prompt revascularization. Number of patients develop new or worsening heart failure (HF) after percutaneous coronary intervention (PCI)-treated STEMI [1]. For example, one contemporary study found that about 3.8% of acute myocardial infarction (AMI) survivors were re-hospitalized for HF within one year [1], and these patients had markedly higher mortality (11.3% vs. 2.5%) [1]. Acute kidney injury (AKI) is also a well-recognized post-STEMI complication, especially after contrast exposure. One large series reported AKI in roughly 7% of STEMI patients undergoing primary PCI [2], rising to ~15% in those ≥75 years [2]. AKI dramatically worsens prognosis: in that cohort AKI was associated with in-hospital death rates of ~27% versus ~2% without AKI [2]. Thus, even sufficient STEMI care leaves appreciable rates of HF admission and AKI, each leading to worse outcome [1, 2].

Given this ongoing risk, there is interest in therapies that might protect the heart and kidneys after STEMI. Sodium–glucose cotransporter-2 (SGLT2) inhibitors are especially attractive candidates. Currently, SGLT2 inhibitors remain indicated in the broader context of secondary prevention and chronic cardiovascular management, but there are currently no guideline/frameworks for initiation during the acute phase of acute coronary syndromes largely due to a lack of robust randomized trials. In chronic HF and chronic kidney disease (CKD) they have proven safety and protective effects, and they work independently of diabetes. Importantly, SGLT2i exert beneficial hemodynamic effects without major hypotension. They produce modest natriuresis and lower systolic blood pressure (SBP) by only ~4–5 mmHg on average [3], and in trials they have not caused excess symptomatic hypotension or need for vasopressors. A recent post-MI trial found no safety concerns when dapagliflozin was started acutely [4], and an empagliflozin trial noted that adverse events matched the known profile with similar rates in drug and placebo groups [5].

SGLT2 inhibitors also have robust renal benefits. In multiple trials of diabetic and non-diabetic kidney disease, they consistently slow CKD progression and reduce albuminuria in patients with or without diabetes [6]. SGLT2 inhibitors restore tubuloglomerular feedback (TGF) by blocking proximal sodium–glucose reabsorption, increasing distal sodium delivery, and constricting the afferent arteriole [7]. This reduces intraglomerular pressure and blunts hyperfiltration, directly protecting damaged nephrons [7]. The resulting natriuresis and modest volume contraction further lower systemic blood pressure and improve renal cortical oxygenation [8]. Altogether, these effects reduce glomerular stress and medullary hypoxia, helping to preserve kidney function during the hemodynamic challenges of acute MI. Beyond hemodynamics, SGLT2 inhibitors augment cardiac metabolism. They raise circulating β-hydroxybutyrate and free fatty acids, providing the myocardium with highly efficient fuels [9]. Clinical data support this: ketone infusion in HF patients improves cardiac output and ejection fraction without increasing oxygen demand [9]. In the acute MI setting, this means SGLT2i-induced hyperketonemia could supply the injured myocardium with an oxygen-sparing energy source, reducing myocardial O_2_ consumption and aiding recovery.

This clinical review uniquely integrates the novel trials with mechanistic rationale. Compared to prior reviews, it highlights that early SGLT2i therapy after STEMI. It will focus exclusively on hemodynamic and renal safety data when initiating SGLT2 inhibitors during STEMI admission*.* We examine whether starting an SGLT2 inhibitor ~24–72 h after PCI in a stable STEMI patient is hemodynamically well tolerated and does not worsen kidney injury. By compiling the latest trial and registry evidence, we seek to inform frontline clinicians about the safety of early SGLT2i initiation in acute MI care.

## 2. Methodology

We performed a narrative literature review using PubMed and Embase from January 2018 to July 2025 with the terms “ST-elevation myocardial infarction”, “acute myocardial infarction”, “SGLT2 inhibitor”, and specific drug names. Additional pivotal trials were identified through a manual search of references and major cardiology conference proceedings. Priority was given to randomized controlled trials (RCTs) and prospective cohorts reporting hemodynamic or renal safety outcomes. Pre-clinical mechanistic studies were included selectively to contextualize physiological effects. Data were extracted qualitatively; no formal meta-analysis was undertaken.

## 3. Mechanistic Rationale

### 3.1 Rapid Natriuresis and Preload Reduction Without Sympathetic Activation

SGLT2 inhibitors block sodium and glucose reabsorption in the proximal tubule, causing glucosuria and natriuresis. This osmotic diuresis lowers plasma and interstitial fluid volume, thereby reducing cardiac preload [10]. For example, empagliflozin reduced blood and plasma volume by about a few percent within two weeks [10]. Dapagliflozin also causes a transient rise in urine sodium and volume, especially in the first few days of therapy [10, 11]. Importantly, these drugs preferentially remove interstitial fluid without sharply depleting intravascular volume [10]. In other words, SGLT2 inhibition may reduce tissue congestion while preserving arterial filling.

Volume reduction with SGLT2 inhibition does not trigger a sympathetic or renin–angiotensin system activation [10, 11]. In animal models, SGLT2 inhibitors even suppress sympathetic nerve activity [11]. The lowered blood pressure with SGLT2i does not raise heart rate [11]. In HF trials, for instance, empagliflozin lowered SBP modestly without causing hypotension or tachycardia [10, 11]. This results in mild unloading of the heart. Cardiac Magnetic resonance imaging (MRI) studies confirm reduced left ventricular end-diastolic volume after SGLT2 therapy, implying lower preload [10]. Thus, SGLT2 inhibitors produce a gentle diuresis and natriuresis that offloads the heart without the usual neurohormonal stress of traditional diuretics [10, 11].

Another consequence is a “diuretic-sparing” synergy when SGLT2i are combined with loop diuretics. Adding an SGLT2 inhibitor in HF patients already on loop diuretics boosts natriuresis beyond either drug alone [11]. This means lower loop doses may achieve similar decongestion, potentially reducing side effects.

### 3.2 Na⁺/H⁺ Exchanger (NHE1) Inhibition and Ketone “Super-Fuel” Shift

In addition, volume effects, SGLT2 inhibitors exert direct metabolic and cellular actions on the heart. One key hypothesis is inhibition of the myocardial Na⁺/H⁺ exchanger (NHE-1). Experimental data show that empagliflozin suppresses cardiomyocyte NHE-1 activity, thus lowering cytosolic Na⁺ and Ca^2+^ concentrations [12]. High intracellular Na⁺ in failing hearts drives calcium overload and impaired relaxation, so SGLT2mediated NHE-1 blockade can improve ion homeostasis. Lower cytosolic Ca^2+^ reduces energy expenditure on ion pumps, while increased mitochondrial Ca^2+^ boosts adenosine triphosphate (ATP) production [12]. In effect, SGLT2 inhibitors may unload the cardiomyocyte energy burden via NHE-1 modulation.

Another major effect is a shift in cardiac fuel utilization toward ketone bodies. SGLT2 inhibitors raise circulating ketone levels by promoting lipolysis and altering the insulin/glucagon ratio [13]. In practice, β-hydroxybutyrate and other ketones double from baseline during sustained SGLT2 therapy [13]. The failing heart can readily oxidize ketones. Indeed, ketone bodies have been termed a “super-fuel” because they produce more ATP per unit oxygen than free fatty acids [13].

The combination of NHE-1 inhibition and increased ketone availability yields a potent energetic benefit. SGLT2 blockade appears to improve mitochondrial function and ATP generation in cardiomyocytes [14, 15, 16]. In one mitochondrial study, SGLT2i elevated coenzyme Q oxidation and enhanced the thermodynamics of ATP hydrolysis [14]. Thus, the heart muscle becomes more efficient. These adaptations may improve cardiac output and myocardial energetics in the acute post-infarction setting [12, 13].

### 3.3 Tubuloglomerular Feedback (TGF) and Intraglomerular Pressure Lowering 

In the kidney, SGLT2 inhibitors trigger TGF that rapidly alters glomerular hemodynamics [17]. By blocking glucose and sodium reabsorption in the proximal tubule, more sodium is delivered to the macula densa [11]. This signals the afferent arteriole to constrict. The net effect is lower intraglomerular pressure and reduced single-nephron glomerular filtration rate (GFR) [7, 11].

Clinically, this mechanism produces a small but immediate estimated glomerular filtration rate (eGFR) decline after starting SGLT2 inhibition. Trials report an average eGFR fall of ~3–6 mL/min/1.73 m^2^ within the first 1–4 weeks [7]. This change is hemodynamic, not nephrotoxic; it reflects the afferent vasoconstriction of TGF. Importantly, once patients are on therapy, GFR stabilizes or even declines more slowly than expected. Long-term, SGLT2 inhibitors slow the rate of kidney function loss, likely by protecting glomeruli from chronic hyperfiltration stress [7].

So, the initial eGFR drop is a reversible adjustment. As pressure inside the glomerulus normalizes, filtration equilibrates. In the context of STEMI and volume management, understanding TGF helps distinguish a harmless SGLT2i-mediated dip from true contrast nephropathy. Overall, TGF explains why SGLT2 inhibitors cause a predictable eGFR dip that usually recovers or stabilizes [7, 11].

To summarize, SGLT2 inhibitors unload the heart by diuresis without reflex neurohormonal activation, reprogram myocardial metabolism for higher efficiency via NHE1 inhibition and ketones, and favorably reset renal hemodynamics through TGF. These mechanisms support early SGLT2i use post-MI by reducing congestion and protecting the kidney and myocardium [7, 10, 12].

### 3.4 Safety Outcomes of SGLT2 Inhibitors in Post-STEMI Randomized Trials

The Dapagliflozin in Myocardial Infarction (DAPA-MI) trial (n = 4017, 2 countries) assessed dapagliflozin in STEMI patients with impaired LV function or Q-wave MI, without diabetes. Patients with eGFR <20 mL/min/1.73 m^2^ or Chronic HF were excluded. Over a median follow-up of 11.6 months, safety was evaluated through serious adverse events leading to hospitalization or treatment discontinuation. Dapagliflozin did not increase rates of adverse events compared with placebo, with particular reassurance regarding hemodynamic stability, renal function, and infections [4].

The Empagliflozin after Acute Myocardial Infarction (EMPACT-MI) trial (n = 6522, 22 countries) tested empagliflozin in patients at high risk of HF after MI. Exclusion criteria included eGFR <20 mL/min/1.73 m^2^ and SBP <100 mmHg, while inclusion of those with eGFR 20–60 mL/min/1.73 m^2^ was added as an enrichment factor along with others. Over a median 17.9-month follow-up, adverse events were assessed broadly, with prespecified interest in AKI, volume depletion, and hypotension. Empagliflozin was well tolerated, with adverse event rates similar to placebo and no signal for renal or hemodynamic instability [5].

The Empagliflozin to prevent worsening of left ventricular volumes and systolic function after myocardial infarction (EMPRESS-MI) trial (n = 105, Austria) studied empagliflozin initiated within 72 h of PCI in STEMI patients. Patients with severe renal dysfunction (eGFR <20 mL/min/1.73 m^2^) or hypotension were excluded. With a median follow-up of 5.5 months, the safety evaluation included prespecified renal and hemodynamic endpoints, as well as adverse events of special interest. No excess in creatinine doubling, AKI, or hypotension was observed compared with placebo, underscoring the renal and hemodynamic safety of early empagliflozin initiation in acute STEMI care [18].

The Empagliflozin in Acute Myocardial Infarction (EMMY) trial (n = 476, UK) also evaluated empagliflozin initiated within 72 h post-PCI in STEMI patients, excluding those with eGFR <45 mL/min/1.73 m^2^ or BP <110/70 mmHg. Over a median follow-up of 6 months, safety endpoints included adverse events leading to discontinuation, renal function decline, and hemodynamic complications. Empagliflozin was associated with no increase in adverse events, with renal and hemodynamic parameters stable relative to placebo [19].

The Empagliflozin Effects in Patients with ST-Elevation Myocardial Infarction (EMI-STEMI) trial (n = 101, single-center, Iran) compared empagliflozin with placebo in STEMI patients without diabetes. Patients with SBP <100 mmHg or eGFR <30 mL/min/1.73 m^2^ were excluded. Over 6 months, adverse events were rare, with no significant differences between treatment groups, suggesting the short-term safety of empagliflozin in this lower-powered study [20]. Summary of these trials is given in Table 1 (Ref. [4, 5, 18, 19, 20]).

**Table 1.** **Safety summary of post-STEMI SGLT2i trials**.

Study	n	Start time post-PCI/MI	Hypotension/BP change (mmHg)	eGFR (Baseline → Change) (mL/min/1.73 m^2^)	AKI	DKA/Genital infections	Follow-up duration (median)	Key takeaway (safety)	Notes (ROB2/Limitations)
DAPA-MI [4]	4017 (2019 vs 1998)	≤10 d (hemodynamically stable) *	NR	Baseline: 83.5 ± 17.1 vs 83.4 ± 16.9; Δ30 w: −4 (IQR −12 to 2) vs −4 (−11 to 3)	NR	No DKA or genital infection reported	11.6 months	Early initiation showed no hemodynamic/renal safety signal in low-risk patients without diabetes/HF	ROB2: Low, Registry-based, low-risk; no acute renal/BP monitoring; hierarchical endpoints not safety-focused; possibly underpowered for rare AEs
EMPACT-MI [5]	6522 (3260 vs 3262)	≤14 d †	Hypotension NR; volume depletion 1.1% vs 1.2%	Baseline stratified by eGFR; no acute dip signal reported	AKI (MedDRA): 0.8% vs 1.3%; CI-AKI: (Creatinine rise >0.5 mg/dL or >25% within 72 h of contrast) 0.2% vs 0.3%; ARF: 1.3% vs 1.8%	DKA: 2/3234 vs 1/3229; genital infections NR; “no increase in class-related AEs”	17.9 months	Safety similar to placebo across risk strata; no hypotension signal	ROB2: some concerns, Endpoints not centrally adjudicated; outpatient HF events excluded; under-representation of women/minorities; focused safety capture
EMMY [19]	476 (237 vs 239)	≤72 h after PCI *	Hypotension NR; inclusion required ≥110/70; no between-group BP concerns reported	Baseline median 92 (IQR 78–102); no significant renal change reported	Renal injury (>2×Cr): 0 vs 0	DKA: 0; Genital fungal: 7/237 vs 2/239; UTI: 11/237 vs 7/239	5.98 months	Early empagliflozin improved NT-proBNP/LV structure-function without significant safety differences	ROB2: Low, Small, not powered for hard endpoints; local echo/labs; limited diversity, under representation of women and diabetics
EMI-STEMI [20]	101 (50 vs 51)	Before PCI; continued 40 d *	No hypotension observed; longitudinal BP change NR	Baseline: 78.4 ± 19.8 vs 77.4 ± 17.4; acute eGFR dip NR	CI-AKI: 4/50 vs 4/51 (*p* = 1.00); KDIGO not specified	DKA: NR; metabolic acidosis none; UTI: 2/50 vs 0/51	6 months	Well tolerated in pre-PCI initiation; renal safety comparable to placebo	ROB2: Low, Small, single-centre; STEMI, non-DM, non-HF; infarct size by cTnI (no MRI); short follow-up; ∼5% LTFU; possible delayed PK effects
EMPRESS-MI [18]	105 (51 vs 54)	12 h–14 d (median 3 d); 1st dose ≤24 h from baseline MRI *	Hypotension NR; no SBP difference through 24 weeks	Baseline: 78.5 ± 20.5 vs 79.3 ± 20.2; Δ24 w: −4.8 vs −7.1 (NS)	KDIGO NR; worsening renal function (≥2×Cr): 1 (placebo)	DKA: none; Genital infections: 3/51 vs 1/54 (2 drug → stop)	5.53 months	No significant between-group safety differences over 24 weeks	ROB2: Low. Small n; short follow-up; mostly STEMI (≈88%) and male (87%); excluded AF/device pts; high background therapy; possible myocardial stunning; limited generalizability

*: Treatment started during hospitalization for acute myocardial infarction, †: Initiation timing unclear: Could be in-hospital or early outpatient depending on discharge. NR, Not reported; n, sample size; IQR, interquartile range; PCI, percutaneous coronary intervention; BP, blood pressure; SBP, systolic blood pressure; CI-AKI, contrast-induced acute kidney injury; ARF, acute renal failure; DKA, diabetic ketoacidosis; eGFR, estimated glomerular filtration rate; Cr, creatinine; AE, adverse events; HF, heart failure; DM, diabetes mellitus; AF, atrial fibrillation; NT-proBNP, N-terminal pro-B-type natriuretic peptide; LV, left ventricle; cTnI, cardiac troponin I; KDIGO, Kidney Disease: Improving Global Outcomes; MRI, magnetic resonance imaging. Dapagliflozin in Myocardial Infarction (DAPA-MI) trial, Empagliflozin after Acute Myocardial Infarction (EMPACT-MI) trial, Empagliflozin to prevent worsening of left ventricular volumes and systolic function after myocardial infarction (EMPRESS-MI) trial, Empagliflozin in Acute Myocardial Infarction (EMMY) trial, Empagliflozin Effects in Patients with ST-elevation myocardial infarction (EMI-STEMI) trial.

Taken together, these five randomized trials consistently demonstrate the safety of initiating SGLT2 inhibitors in the acute and subacute STEMI setting. Across multinational mega-trials (DAPA-MI, EMPACT-MI) and smaller studies (EMPRESS-MI, EMMY, EMI-STEMI), adverse event rates were comparable to placebo. Importantly, renal and hemodynamic outcomes showed no excess risk, even when therapy was initiated within 72 h of PCI. Events such as AKI, hypotension, or volume depletion were infrequent and not significantly different between groups. Infectious complications, including urinary tract or genital infections, were uncommon and did not exceed placebo rates. Similarly, metabolic adverse events such as ketoacidosis or hypoglycemia were not observed in excess. Mortality outcomes were comparable across arms, reinforcing the neutral-to-reassuring safety profile. Collectively, these findings suggest that early use of SGLT2 inhibitors post-STEMI is feasible and well tolerated in carefully selected patients. Nevertheless, the exclusion of those with severely impaired renal function or significant hemodynamic instability indicates that results should not be generalized to the most vulnerable post-MI populations. In summary, the uniformity of safety signals across trials strengthens confidence in therapeutic use and directs attention toward evaluating efficacy in diverse, real-world patient groups.

## 4. Safety Considerations

Starting SGLT2 inhibitors during STEMI admission requires a careful balance between capturing their cardioprotective and renoprotective benefits and avoiding complications in the acute phase. The following safety domains are important in informing bedside decisions.

### 4.1 Hemodynamic Thresholds: When Not to Start

A key prerequisite for starting SGLT2 inhibitors during STEMI is ensuring hemodynamic stability. Key eligibility criteria include a SBP of at least 100 mmHg and a Killip class ≤II, with no ongoing escalation of inotropes or vasopressors, as these indicate underlying instability. In patients with borderline SBP (e.g., 90–100 mmHg), initiation should not be automatically pursued or abandoned; instead, temporary withholding or dose reduction of loop diuretics may be considered to minimize volume-related hypotension, with reassessment after 12–24 h once hemodynamic parameters have stabilized. Before initiation, the absence of orthostatic hypotension or postural symptoms should be confirmed. Avoiding hypotensive episodes after starting therapy is critical, as even transient drops in blood pressure in the setting of an infarcted myocardium may exacerbate ischemia or delay recovery [12, 21, 22, 23].

### 4.2 Renal Function and eGFR ‘Dip’: What is Benign

An early and well-recognized effect of SGLT2 inhibitor therapy is the initial decline in eGFR, typically within 10–20% of baseline, occurring within days of initiation. This change is predominantly hemodynamically mediated via TGF, whereby afferent arteriolar vasoconstriction decreases intraglomerular pressure and mitigates hyperfiltration [12, 24]. The initial decline is considered benign, as it usually stabilizes within 1–2 weeks, and is usually unaccompanied by structural renal injury [21], and is followed by long-term preservation of renal function, with evidence suggesting that SGLT2 inhibitors slow the progression of CKD [21, 22]. Interestingly, this initial decline does not predict long-term renal function loss. Nevertheless, in patients with suspected or evolving AKI, especially those meeting Kidney Disease: Improving Global Outcomes (KDIGO) stage 1 or higher, initiation should be deferred until renal function has stabilized. Furthermore, therapy should be temporarily withheld if serum creatinine rises by more than 30% from baseline within the first week or if there is ongoing volume depletion, such as from vomiting, diarrhea, or excessive diuresis [23].

Many common precipitants of AKI have no direct mechanistic relationship to SGLT2 inhibitor therapy, including conditions such as sepsis, non-obstructive post-renal causes, or anaphylaxis. In contrast, certain AKI risk factors may be favorably influenced by SGLT2 inhibition—through improvements in CKD progression, HF status, and glycemic control—while others may be exacerbated, particularly volume depletion or ketoacidosis. Accordingly, initiation of SGLT2 inhibitors in patients with markedly reduced GFRs is generally discouraged because of the anticipated early decline in GFR. Moreover, temporary cessation of therapy is advised during episodes of acute renal dysfunction, hypovolemia, or hemodynamic instability to minimize potential renal risk.

### 4.3 Contrast Nephropathy: Procedural Context Matters

PCI and diagnostic angiography expose patients to contrast media, which is a known contributor to contrast-associated AKI (CA-AKI). It is also worth mentioning that SGLT2 inhibitors may confer nephroprotection against contrast-induced acute kidney injury (CI-AKI), a major complication of iodinated contrast exposure [25]. Systematic reviews and meta-analyses have shown that chronic SGLT2 inhibitor use is associated with a significant reduction in the incidence of CI-AKI, with reported risk reductions up to approximately 48–63% compared with non-use in type 2 diabetes cohorts undergoing coronary angiography or PCI [25]. This protective effect is thought to derive from favorable hemodynamic modulation—including reduced glomerular hyperfiltration, lowered intraglomerular pressure, improved renal perfusion, and anti-inflammatory/antioxidant actions—which together mitigate tubular stress and oxidative injury following contrast administration [25, 26]. Although these findings are promising, the evidence base remains largely observational, and prospective RCTs are needed to confirm efficacy, clarify optimal timing/duration, and establish these agents within CI-AKI preventive strategies.

To reduce the risk of contrast-associated AKI, SGLT2 inhibitor initiation should ideally be delayed for at least 24–48 h following PCI, particularly in patients with borderline renal function or those who have undergone multiple contrast exposures. Adequate pre- and post-procedural hydration, preferably with isotonic saline, is essential to maintain renal perfusion [27]. Furthermore, concurrent use of non-steroidal anti-inflammatory drugs (NSAIDs) and nephrotoxic antimicrobials should be avoided to further reduce renal injury risk [25, 28].

### 4.4 Rare but Serious Events: DKA and Genital Infections

SGLT2 inhibitors have been associated with euglycemic diabetic ketoacidosis (euDKA), a rare but potentially life-threatening complication that can develop in the peri-infarct period or during prolonged fasting. Risk factors for euDKA include type 1 diabetes mellitus, where SGLT2 is contraindicated, recent fasting or compliance to ketogenic diets, perioperative stress, and insulin deficiency or omission. At discharge, patients should be counselled on sick-day rules, including the need to withhold SGLT2i during episodes of acute illness, vomiting, or reduced oral intake, and to seek immediate medical attention if symptoms suggestive of DKA occur. Another recognized adverse effect of SGLT2 inhibitors is genital mycotic infection—a consequence of glucosuria creating a favorable environment for pathogenic fungal growth [29]. Clinical data indicate that the risk of genital infections increases approximately 2- to 6-fold among SGLT2i users compared to those not using these agents [29]. Fortunately, most of these infections are mild to moderate in severity and respond well to standard antifungal therapy; routine discontinuation of SGLT2i is not typically required [30].

## 5. The START Algorithm

To support frontline clinicians, we propose the START algorithm, a practical bedside tool for guiding SGLT2i initiation during STEMI admission [31, 32]. Each letter corresponds to a safety checkpoint.

### 5.1 S — Stable Hemodynamics

For inclusion, the patient was required to have an SBP of at least 100 mmHg, ideally measured in both the lying and standing positions. The Killip class was limited to I or II, indicating the absence of pulmonary edema or overt HF. There should have been no escalation in the use of inotropes or vasopressors within the preceding 12–24 h. Additionally, patients were required to maintain an oxygen saturation of ≥92% on room air or remain stable on low-flow oxygen support. The ability to tolerate oral medications was also a prerequisite [22].

### 5.2 T — Tubular Reserve

The renal (tubular reserve) criteria required an eGFR of at least 25 mL/min/1.73 m^2^. Creatinine trends were reviewed, with stability over 24–48 h considered indicative of adequate renal reserve. Initiation was deferred in the presence of a creatinine rise of ≥0.3 mg/dL, KDIGO stage 1 or higher AKI, or recent contrast exposure associated with renal deterioration. Patients with known CKD stage 3b were still considered eligible; however, they required close monitoring [22, 33].

### 5.3 A — Acid–Base Status

Metabolic stability was confirmed by ensuring the absence of ongoing acidosis, defined as a serum bicarbonate level of ≥22 mmol/L, a normal anion gap, and no significant urinary or serum ketones. Use was avoided in patients adhering to ketogenic diets or undergoing prolonged fasting, as well as in those with a recent history of diabetic ketoacidosis (DKA) or poorly controlled glycemia [34].

### 5.4 R — Risk Factors

Contraindications to in-hospital initiation included type 1 diabetes mellitus, recent DKA (within the past three months), and a perioperative state such as recent coronary artery bypass grafting (CABG). Additional exclusion criteria were severe dehydration, significant gastrointestinal losses, or hypotensive volume depletion [34], as well as concurrent SGLT2 inhibitor therapy without adequate monitoring. Patients meeting these criteria were excluded from initiation during hospitalization and were advised to be reassessed at a later stage.

### 5.5 T — Timing

The optimal window for initiation was 24–72 h PCI, provided hemodynamic stability had been achieved, diuretic and ACE inhibitor/ARB/mineralocorticoid receptor antagonist (MRA) therapy had been reconciled, and there was no evidence of active renal decline [22, 31, 32]. Coordination with the cardiology, nephrology, and diabetes teams was recommended to ensure safe and effective implementation.

The following “START” checklist (Table 2) may assist clinicians in deciding whether to initiate an SGLT2 inhibitor during the index STEMI admission. It was derived from inclusion/exclusion criteria of contemporary randomized trials (DAPA-MI, EMPACT-MI, EMMY, EMPRESS-MI) and augmented by expert clinical judgment. Because large outcome studies such as EMPA-STEMI and the follow-up phase of EMPRESS-MI are still in progress, the thresholds below remain interim guidance rather than a formal guideline recommendation. These criteria are intended to guide, not dictate, practice and should be re-evaluated once the results of ongoing trials are published and incorporated into professional society statements.

**Table 2.** **START checklist**.

Letter	Safety Check	Pragmatic Threshold	Primary Trial Precedent/Evidence	Additional Rationale
S	Stable hemodynamics	SBP ≥100 mmHg; Killip ≤II; no inotropes/vasopressors within 24 h	DAPA-MI, EMPACT-MI	Lower values were explicit exclusion criteria in both trials; they preserve coronary perfusion pressure.
T	Tubular reserve	eGFR ≥25 mL/min/1.73 m^2^ AND <30% creatinine rise over 48 h	EMPRESS-MI (≥20); EMMY (≥45)	Below 25, the hemodynamic eGFR dip is harder to separate from early AKI.
A	Acid–base stability	Serum HCO_3_⁻ ≥22 mmol/L, normal anion gap, negative/trace ketones	No RCT mandate; expert consensus based on DKA case series	Minimizes risk of euglycemic DKA during acute stress.
R	Risk factors/contra-indications	Exclude: type 1 DM, recent DKA (<3 mo), cardiogenic shock, peri-operative status	Uniform across all five trials	Situations with unpredictable volume shifts or insulin deficiency.
T	Timing window	24–72 h after successful PCI, once hemodynamics are stable	EMMY (≤72 h), EMPRESS-MI (median 3 d)	Allows contrast wash-out and initial renal trend assessment.

How to use: Tick all five boxes before the first dose. Re-check vitals q8–12 h for 48 h; repeat creatinine/ketones at 48–72 h. Level of evidence: Ties directly to inclusion/exclusion thresholds of ≥5 contemporary RCTs (see Table 1); no randomized data yet in Killip III–IV, SBP <100 mmHg, or eGFR <20 mL/min/1.73 m^2^. eGFR, estimated glomerular filtration rate; SBP, systolic blood pressure; DM, diabetes mellitus; DKA, diabetic ketoacidosis; AKI, acute kidney injury; RCTs, randomized controlled trials. Dapagliflozin in Myocardial Infarction (DAPA-MI) trial, Empagliflozin after Acute Myocardial Infarction (EMPACT-MI) trial, Empagliflozin to prevent worsening of left ventricular volumes and systolic function after myocardial infarction (EMPRESS-MI) trial, and Empagliflozin in Acute Myocardial Infarction (EMMY) trial.

### 5.6 Monitoring Plan

Monitoring included assessment of vital signs, with blood pressure and heart rate measured every 8–12 h during the first 48 h, and vigilance for postural changes or symptomatic hypotension [33, 35]. Laboratory evaluation comprised creatinine and eGFR measurement at 48–72 h and again at 1–2 weeks, with serum ketones checked if symptoms suggestive of acidosis emerged [34]. Drug interaction precautions involved monitoring for volume depletion when using loop diuretics, with dose reduction if orthostatic symptoms developed [31, 36]. Avoidance of concurrent NSAID use and patient education on sick-day rules prior to discharge [37].

## 6. Future Directions and Research Gaps

Although the rationale for early initiation of SGLT2i in STEMI is strong, several important uncertainties remain. Large-scale clinical trials are currently underway to address these questions. The EMPA-STEMI trial is evaluating the early use of empagliflozin in STEMI patients following PCI, with a focus on myocardial salvage and ventricular remodeling outcomes [20]. Similarly, the EMPRESS-MI trial is investigating in-hospital SGLT2 initiation in post-MI patients with diabetes, assessing its impact on cardiac function recovery and renal outcomes [38]. Findings from these studies are expected to clarify safety, efficacy, and optimal timing of therapy in this high-risk population.

Several evidence gaps persist. Patients with cardiogenic shock and STEMI remain excluded from current trials, leaving the safety profile in this subgroup unknown. Similarly, data are limited on initiation in individuals with advanced CKD stage 4–5, particularly regarding AKI risk. In addition, the role of invasive hemodynamic monitoring, such as right heart catheterization, in identifying patients most likely to tolerate and benefit from SGLT2i therapy has yet to be defined. Addressing these gaps will be crucial to optimizing patient selection, refining initiation protocols, and safely integrating SGLT2 into comprehensive post-STEMI management strategies.

## 7. Conclusion

In patients hospitalized with STEMI, the judicious initiation of SGLT2i presents a promising strategy to mitigate cardiorenal complications. When commenced 24–72 h post-PCI in clinically stable patients, existing evidence indicates no consistent safety concerns regarding hypotension, AKI, or DKA. The early decline in eGFR observed with therapy initiation is typically mild, predictable, and self-limiting. Adapting a structured, safety-focused framework such as the START algorithm enables clinicians to identify suitable candidates, reduce complications, and implement optimal monitoring. The dual cardiovascular and renal benefits of SGLT2 inhibitors further underscore their therapeutic potential in post-myocardial infarction care. Emerging data support in-hospital initiation in hemodynamically stable patients, but routine incorporation into STEMI pathways awaits confirmation from ongoing outcome trials that will refine the understanding of optimal timing, safety profile, and long-term outcomes.
